# Repressive C2H2 zinc finger ZAT proteins promote programmed cell death in the Arabidopsis columella root cap

**DOI:** 10.1093/plphys/kiad130

**Published:** 2023-02-28

**Authors:** Qiangnan Feng, Marta Cubría-Radío, Tereza Vavrdová, Freya De Winter, Neeltje Schilling, Marlies Huysmans, Amrit K. Nanda, Charles W. Melnyk, Moritz K. Nowack

**Affiliations:** 1 Department of Plant Biotechnology and Bioinformatics, Ghent University, 9052 Ghent, Belgium; 2 VIB-UGENT Center of Plant Systems Biology, 9052 Ghent, Belgium; 3 Institute of Biochemistry and Biology, Potsdam University, 14476 Potsdam OT Golm, Germany; 4 Department of Plant Biology, Swedish University of Agricultural Sciences, Uppsala, Sweden

## Abstract

Developmental programmed cell death (dPCD) controls a plethora of functions in plant growth and reproduction. In the root cap of *Arabidopsis thaliana*, dPCD functions to control organ size in balance with the continuous stem cell activity in the root meristem. Key regulators of root cap dPCD including SOMBRERO/ANAC033 (SMB) belong to the NAC family of transcription factors. Here we identify the C2H2 zinc finger protein ZAT14 as part of the gene regulatory network of root cap dPCD acting downstream of SMB. Similar to SMB, ZAT14 inducible misexpression leads to extensive ectopic cell death. Both thecanonical EARmotif and a conserved L-box motif of ZAT14 act as transcriptional repression motifs and are required to trigger cell death.While a single *zat14* mutant does not show a cell death-related phenotype, a quintuple mutant knocking out five related ZAT paralogs shows a delayed onset of dPCD execution in the columella and the adjacent lateral root cap. While ZAT14 is co-expressed with established dPCD-associated genes, it does not activate their expression. Our results suggest that ZAT14 acts as a novel transcriptional repressor controlling a so far uncharacterized sub-section of the dPCD gene regulatory network active in specific root cap tissues.

## Introduction

Developmental programmed cell death (dPCD) is a genetically controlled biological process essential for plant development(Van Durme and Nowack, 2016; Locato and De Gara, 2018). Different dPCD processes occur in a multitude of developmental contexts, including tracheary element differentiation, anther and pollen formation, seed development, and root cap turnover (Daneva et al., 2016; Locato and De Gara, 2018). The root cap is a specialized external organ at the root tip that protects the root apical meristem and acts as a sensory organ to optimize root growth and root system architecture(Kumpf and Nowack, 2015; Xuan et al., 2016; Di Mambro et al., 2018). The root cap of *Arabidopsis thaliana* (Arabidopsis) originates from two distinct stem cell populations: The columella initialsform a plate below the quiescent center whereas the epidermal/lateral root cap (EPI/LRC) initials are arranged in a ring around the columella initials. Iterative formative divisions of columella initials generate new layers of columella cells, while the EPI/LRC initials divide to form LRC and epidermis layers. While columella cells differentiate and expand directly, the LRC cells of the same root cap layer first undergo a number of cell divisions before differentiation to cover the root meristem until the start of the elongation zone (Kumpf and Nowack, 2015; Ganesh et al., 2022). At the start of the elongation zone, the most distal LRC cells of each cell file will undergo a precisely timed dPCD process to ensure that the extend of the LRC is restricted to the meristematic region, which is important for optimal root growth(Fendrych et al., 2014). In contrast, the columella cellsare shed in intervals as packages of living border-likecells (Vicre et al., 2005), and only complete dPCD during and after their release into the rhizosphere (Huysmans et al., 2018).

Transcriptional regulation is crucial for dPCD control in the root cap and other cell types (Cubria-Radio and Nowack, 2019; Jiang et al., 2021). Until now, the NAM ATAF and CUC (NAC) domain transcription factors (TFs) ANAC033/SOMBRERO (SMB), ANAC087 and ANAC046 have been identified as key TFs that orchestrate different aspects of root cap cell death. Moreover, several transcriptionally regulated dPCD-associated genes, including BIFUNCTIONAL NUCLEASE1 (BFN1), RIBONUCLEASE3 (RNS3), and PUTATIVE ASPARTIC PROTEINASE A3 (PASPA3), EXITUS1 (EXI1)are direct or indirect downstream targets of SMB, ANAC087 and ANAC046 (Fendrych et al., 2014; Huysmans et al., 2018). Notably, dPCD-associated genes are upregulated in different tissues undergoing dPCD, suggesting the existence of a core machinery of dPCD in plants(Olvera-Carrillo et al., 2015).

The root cap-specific TF SMB transcriptionally controls the preparation of LRC dPCD, the ultimate stage of their differentiation.Several transcriptionally regulated PCD-associated genes are positioned downstream of SMB since in the *smb* mutant or inducible SMB overexpression (OE) lines, their expression is decreased or increased, respectively (Fendrych et al., 2014; Huysmans et al., 2018). Accordingly, mutating of *SMB* leads to a delay or absence of root cap cell death (Fendrych et al., 2014), while its inducible systemic misexpression causes plant-wide ectopic cell death leading to growth arrest and eventually death of the plant (Bennett et al., 2010; Fendrych et al., 2014; Huysmans et al., 2018).

We hypothesized that the PCD gene regulatory network contains additional transcriptional regulators, including transcriptional repressors, to ensure a tight and failsafe control of root cap maturation, dPCD preparation and execution, as well as post-mortem corpse clearance. Here we identify ZINC-FINGER OF ARABIDOPSIS THALIANA14 (ZAT14)(Ciftci-Yilmaz and Mittler, 2008) and related ZAT TFs as part of the SMB-controlled gene regulatory network of root cap cell death. The C2H2-type zinc finger protein family includes 176 members in Arabidopsis, being one of the largest families of putative transcriptional regulators in plants (Englbrecht et al., 2004; Ciftci-Yilmaz and Mittler, 2008; Xie et al., 2019). ZAT14 and several related ZAT proteins belong to the C2H2 C1-2i subclass containing 20 members, while other ZAT proteins fall into the related C1-3i subclass containing 8 members(Ciftci-Yilmaz and Mittler, 2008). Several ZATshave been shown to play important roles in plant stress responses by transcriptional regulation (Xie et al., 2019), but most of the ZAT proteins remain functionally uncharacterized.

Inducible ZAT14 misexpression mimics the ectopiccell death phenotype of SMB misexpressors, and SMB promotes ZAT14 expression. Single-cell RNA-sequencing (scRNA-seq) data suggest that ZAT14 is coexpressed with established dPCD-associated genes in the root cap and root xylem, which we confirmed by transcriptional and translational reporter lines. Protein domain analyses show that ZAT14 acts as a transcriptional repressor and that mutating repressive motifs interferes with ZAT14’s potential to induce cell death. Transcriptome analyses show that SMB and ZAT14 misexpression cause downregulation of a common set of genes which have not been implicated in PCD so far. Though a *zat14*single mutant does not show any PCD-related phenotypes, higher-order mutants in related *ZAT*genes show a delay in columella cell death placing repressive ZAT TFs on a hitherto unrecognized branch of the root cap PCD gene regulatory network downstream of SMB.

## Results

### TRANSPLANTA screening to identify additional regulators of dPCD

**Figure 1:**
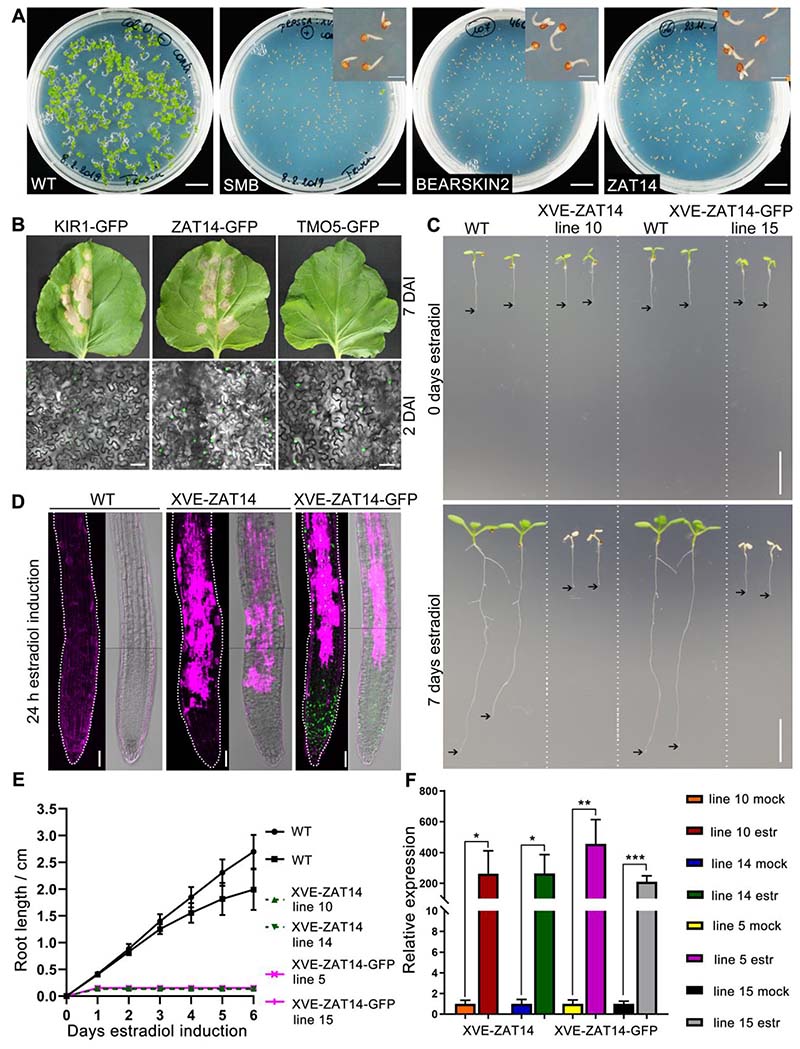
Overexpression of ZAT14 in *N. benthamiana* leaves and in Arabidopsis seedlings. (A) TRANSPLANTA screening. Seeds of lines overexpressing SMB, BEARSKIN 2 and ZAT14 TFs were germinated on medium containing 10 µM estradiol for 10 days, while the wild-type (WT) was used as a negative control. Bars = 1 mm for the insert images, 1 cm for the rest. (B) Overexpression of ZAT14 in *N. benthamiana* leaves. The leaves expressing *pro35S:KIR1-GFP*, *pro35S:ZAT14-GFP* and *pro35S:TMO5-GFP* were imaged at 7 days after infiltration (DAI). *pro35S:KIR1-GFP* was used as a positive control, *pro35S:TMO5-GFP* was used as a negative control. GFP signal was imaged in infiltrated *N. benthamiana* leaves at 2 DAI. The representative pictures of at least three biological replicates are shown here. Bars = 50 µm. (C) Macroscopic images of WT seedlings and representative estradiol-inducible *proHTR5:XVE>>ZAT14* and *proHTR5:XVE>>ZAT14-GFP* lines at 0 and 7 days after transfer to estradiol-containing medium. Black arrows point at root tips. Bars = 1 cm. (D) Confocal laser scanning micrograph (CLSM) of roots inducibly overexpressing ZAT14 and ZAT14-GFP 24 hours after estradiol induction were stained with the propidium iodide (PI). PI is shown in magenta and GFP is in green. Each panel consists of two stitched images. White dotted lines mark the root profile. Bars = 50 µm. (E) Quantification of the Arabidopsis root growth of two independent lines of ZAT14 and ZAT14-GFP overexpressing lines. At least 10 roots of each line were quantified. Results shown are means ± SD. (F) As indicated by RT-qPCR analysis, 8 hours after the induction by estradiol (estr) leads to strong expression of ZAT14 in two independent lines of XVE-ZAT14 and XVE-ZAT14-GFP compared to the mock. Results shown are means ± SD (three independent treatments and three technical repeats each). Statistical analysis was performed by *t* test; * p < 0.05, ** p < 0.01, *** p < 0.001. See also Supplemental movies 1 and 2.

In order to find additional TFs of the dPCD gene regulatory network, we screened a subset of root-cap expressed TFs of the TRANSPLANTA (TPT) seed collection. The TPT collection consists of homozygous Arabidopsis lines each expressing one TF under the control of a systemic β-estradiol-inducible promoter(Coego et al., 2014).We screened for TFs which upon induction by estradiol treatment mimicked the SMB misexpression phenotype, which is characterized by ectopic cell death followed by growth arrest and plant death (Bennett et al., 2010; Fendrych et al., 2014; Huysmans et al., 2018). When *proRPS5A:XVE>>SMB-GFP* seeds are germinated on medium containing estradiol, their roots emerge but rapidly stop growing due to ectopic cell death and the cotyledons do not emerge from the seed coat (Figure 1A).

We did not screen the entire collection but filtered for root-cap expressed and PCD-associated TFs using a publicly available scRNA-seq dataset(Ferrari et al., 2022).Of the 650 TFs included in the collection, 320 TFs qualified as expressed in the lateral root cap (LRC) and columella clusters. When further filtering for TFs expressed in the dPCD population (clusters in which*PASPA3*, *BFN1* and *RNS3* are highly expressed), we identified 57 TFs, expressed by a total of 150independent TPT lines. These lines were screened for a SMB-like phenotype as described above (Supplemental dataset 1). We used *proRPS5A:XVE>>SMB-GFP* seeds as a positive control and Col-0 seeds as a negative control. We only identified two TF genesshowing an SMB-like misexpression phenotype: one line misexpressing *BEARSKIN2* (*BRN2*, AT4G10350) and three lines misexpressing *ZINC-FINGER OF ARABIDOPSIS THALIANA 14* (*ZAT14*, AT5G03510) (Figure1A). As *BRN2* has already been shown to induce ectopic cell death (Bennett et al., 2010), we focused on *ZAT14* which has been implicated in phloem differentiation but not in dPCD(Roszak et al., 2021).

### *ZAT14* overexpression is sufficient to induce ectopic cell death

To independently test the effect of ZAT14 misexpression we first used transient transfection of *Nicotiana benthamiana*. As expected for a TF, a *pro35S:ZAT14-GFP* construct conveyed ZAT14-GFP localization in the nucleus 2 days after infiltration (DAI, Figure 1B). At 7 DAI *ZAT14-GFP*expression induced a macroscopically visible tissue degeneration phenotype. This phenotype was characterized by chlorosis and extensive leaf tissue death (Figure 1B), and was comparable to the phenotype generated by the expression of an established dPCD-promoting gene, *pro35S:KIR1-GFP*(Figure 1B) (Gao et al., 2018). Conversely, the expression of TARGET OF MONOPTEROS 5 (TMO5/BHLH32), a TF not associated with cell death, did not lead to chlorosis. In this experimental setup, TMO5 serves as a negative control (Figure 1B).

To confirm the TPT results in Arabidopsis, we generated vectors conveying estradiol-inducible (Siligato et al., 2016) ZAT14 expression controlled by the ubiquitous promoter *proHTR5*(Ingouff et al., 2017)(Figure 1C-E, Supplemental movie 1).In 35 independent linesexpressing ZAT14 either with or without C-terminal GFP tag for protein visualization, the overexpression of ZAT14 first triggered root growth arrest24 hours after transfer to estradiol-containing medium(Figure 1E, Supplemental figure 1B), and later led to the death of the entire seedling (Figure 1C). To visualize cell death in roots, we used the membrane-impermeable dye propidium iodide (PI). PI entry into the cell indicates plasma membrane permeabilization as a central cell death hallmark(Truernit and Haseloff, 2008; Fendrych et al., 2014). 24 hours after induction (HAI), root cells in the transition and elongation zone are already intensely stained by PI indicating widespread cell death in this part of the root (Figure 1D), explaining the macroscopically visible root growth arrest. This ectopic cell death phenotypeis reminiscent of inducible misexpression of SMB, NAC046, ANAC087, and KIR1(Gao et al., 2018; Huysmans et al., 2018). Expression analysis using RT-qPCR confirmed that *ZAT14* was already strongly up-regulated at 8HAI (Figure 1F). Microscopic time-lapse imaging of *proHTR5:XVE>>ZAT14-GFP*roots, and *proHTR5:XVE>>NLS-GFP*control roots, revealed GFP expression 6HAI (Supplemental movies1 and 2), and first cases of ectopic cell death caused by ZAT14-GFP as early as 10 HAI (Supplemental movie 1).Our results showthat inducible misexpression of ZAT14 is sufficient to trigger ectopic cell death outside the root cap context.

### L-box and EAR domain of ZAT14 are required to cause ectopic cell death

ZAT14 belongs to theC1-2i subclass of C2H2-type zinc finger proteins which contains a total of 20 members (Ciftci-Yilmaz and Mittler, 2008). There are several conserved regions present in the majority of C1-2i members: A short motif including a consensus sequence corresponding to a B-box (KXKRSKRXR) is located near the N-terminus and might act as a nuclear localization signal(Sakamoto et al., 2000). The second motif consists of acidic residues followed by hydrophobic leucine rich residues, with a consensus of “EXEXXAXCLXXL” (L-box), which is located between the B-box and the first zinc finger domain. Furthermore, there are two C2H2 zinc finger domains mediating interaction with DNA, and at the C-terminus, there is an ethylene-responsive element binding factor (ERF)-associated amphiphilic repression (EAR) motif(Supplemental figure 1A) (Sakamoto et al., 2000; Englbrecht et al., 2004; Ciftci-Yilmaz and Mittler, 2008; Xie et al., 2019). Thetranscriptional repression function of several C2H2 TFs has been attributed to regions containing an EAR motif(Ohta et al., 2001; Hiratsu et al., 2002).Together with the EAR-motif, the L-box may play roles in protein-protein interactions important for gene repression(Sakamoto et al., 2000), but experimental evidence to support this is lacking so far.

**Figure 2:**
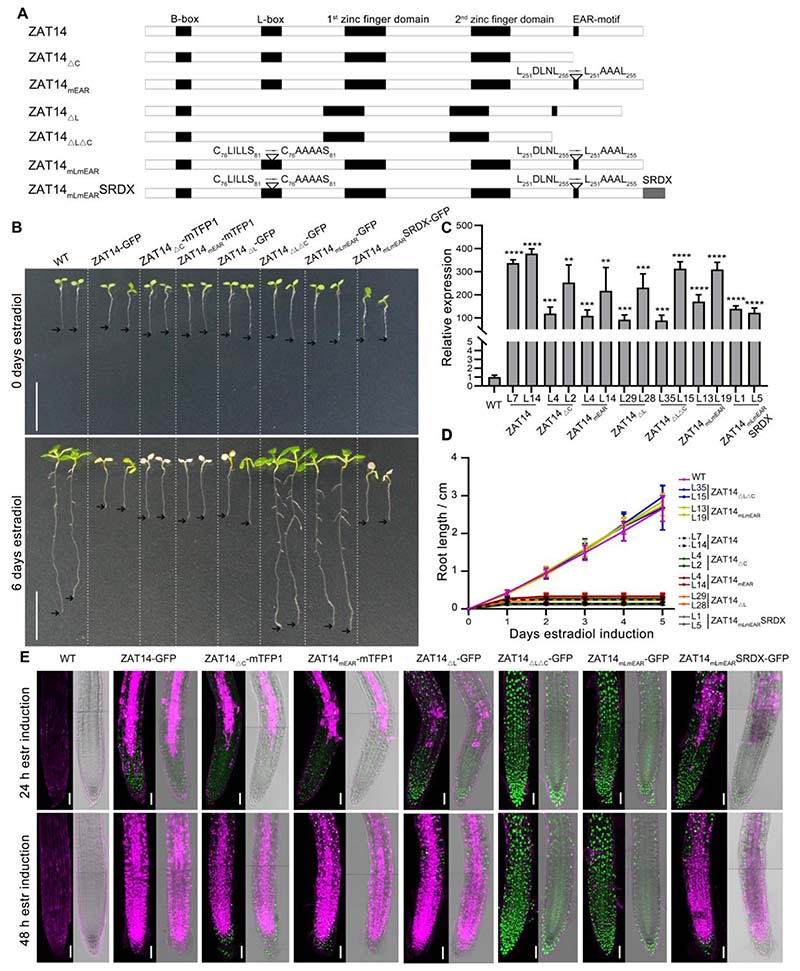
L-box and EAR domain of ZAT14 are required to cause ectopic cell death. (A) Graphical representation of ZAT14 modified versions. Full length ZAT14 has a B-box, an L-box, two zinc finger domains, and an EAR-motif. ZAT14_ΔC_ has 41 deleted amino acids in the C-terminal in which the EAR-motif domain is included. ZAT14_mEAR_ has a mutated EAR-motif. In ZAT14_ΔL_, the L-box (69aa-80aa) has been deleted. In ZAT14_ΔLΔC_, the L-box domain as well as the C-terminal were deleted. In the ZAT14_mLmEAR_, the L-box domain and the EAR-motif domain were mutated. In the ZAT14_mLmEAR_SRDX, the SRDX motif was added at the C terminal of ZAT14_mLmEAR_. (B) Macroscopic appearance of WT seedlings and representative estradiol-inducible ZAT14 modified versions at 0 and 6 days after transfer to estradiol-containing medium. Overexpression of the ZAT14 wild type, ZAT14_ΔC_, ZAT14_mEAR_, ZAT14_ΔL_ and ZAT14_mLmEAR_SRDX resulted in root growth arrest. Similar to the wild type seedlings, the overexpression of ZAT14_ΔLΔC_ and ZAT14_mLmEAR_ did not result in root growth arrest after 6 days of estradiol induction. All these constructs were driven by HTR5 promoter. Black arrows point at root tips. Bars = 1 cm. (C) As indicated by RT-qPCR analysis, 8 hours induction by estradiol caused strong expression of ZAT14 in two independent lines of each construct compared to wild type. Results shown are means ± SD (three independent treatments and three technical repeats each). Statistical analysis was performed by *t* test; ** p < 0.01, *** p < 0.001, **** p < 0.0001. (D) Quantification of the Arabidopsis root growth of two independent lines of each modified ZAT14 overexpressing lines. At least 10 roots of each line were quantified. Results shown are means ± SD. (E) Overexpression of the ZAT14 wild type, ZAT14_ΔC_, ZAT14_mEAR_, ZAT14_ΔL_ and ZAT14_mLmEAR_SRDX resulted in ectopic cell death in the root of Arabidopsis, stained with PI (in magenta), after 24h and 48h of estradiol treatment. The overexpression of ZAT14_ΔLΔC_ or ZAT14_mLmEAR_ did not result in aberrant cell death. mTFP1 and GFP fused to ZAT14, in green. The fluorescent signal is shown as a Z-projection, whereas the brightfield channel is shown as a single stack. Each panel consists of two stitched images. Bars = 50 μm.

To study the importance of the differentconservedZAT14 domains contribute to the misexpression phenotype, we made different mutated versions of ZAT14. First, we deleted the 41 C-terminal amino acids containing the EAR-motif (ZAT14_ΔC_), or mutated the EAR-motif modifying L_251_DLNL_255_ to L_251_AAAL_255_ (ZAT14_mEAR_)(Ciftci-Yilmaz et al., 2007). Next, we deleted the L-box (ZAT14_ΔL_) and ultimately, we deleted (ZAT14_ΔLΔC_) or mutated (ZAT14_mLmEAR_) both domains simultaneously. In the ZAT14_mLmEAR_ version, the L-box was mutated modifying C_76_LILLS_81_ to C_76_AAAAS_81_ and the EAR-motif was mutated as described above (Figure 2A). We introduced vectors for estradiol-inducible overexpression of all different ZAT14 versions C-terminally fused to either GFP or mTFP1 (Pasin et al., 2014)into Arabidopsis, establishing at least 20 independent lines for each construct for phenotypic analysis. In summary, we found that only joint mutation or deletion of both L-box and EAR-motif compromised the pro-PCD function of ZAT14 (Supplemental figure 1B). To maximize comparability between different lines we performed qPCR and picked two lines per construct with about 100-fold and 300-fold upregulation in comparison to the wild type (Figure 2C).We found the ZAT14 wild-type protein, ZAT14_mEAR_-mTFP1, ZAT14_ΔC_-mTFP1, and ZAT14_ΔL_-GFP caused root growth arrest (Figure 2B, D) and rapid cell death (Figure 2E) upon induction. However, overexpression of ZAT14_ΔLΔC_-GFP or ZAT14_mLmEAR_-GFP did not result in root growth arrest (Figure 2B, D) or ectopic cell death (Figure 2E). To test whetherZAT14 acts via gene repression, we added the repressive SRDX motif (Hiratsu et al., 2003) to the C-terminus of ZAT14_mLmEAR_. Two independent lines were selected based on the expression level via qPCR, and both showed a reconstitution of ZAT14’s pro-PCD function to the wild-type level (Figure 2 B, D and E, Supplemental figure 1B).Taken together, these results suggest that ZAT14 promotes ectopic cell death by the mechanism of gene repression, and that both the EAR-motif and the L-box are important for this repressive function.

### ZAT14 is highly upregulated prior to dPCD processes in the xylem and the root cap

Available scRNAseq dataset of Arabidopsis roottips (Ferrari et al., 2022) show that *ZAT14* is not only upregulated prior to root cap dPCD, but also during protoxylem maturation and thus co-expressed with canonical dPCD associated genes (Olvera-Carrillo et al., 2015) (Supplemental figure 2A).

**Figure 3:**
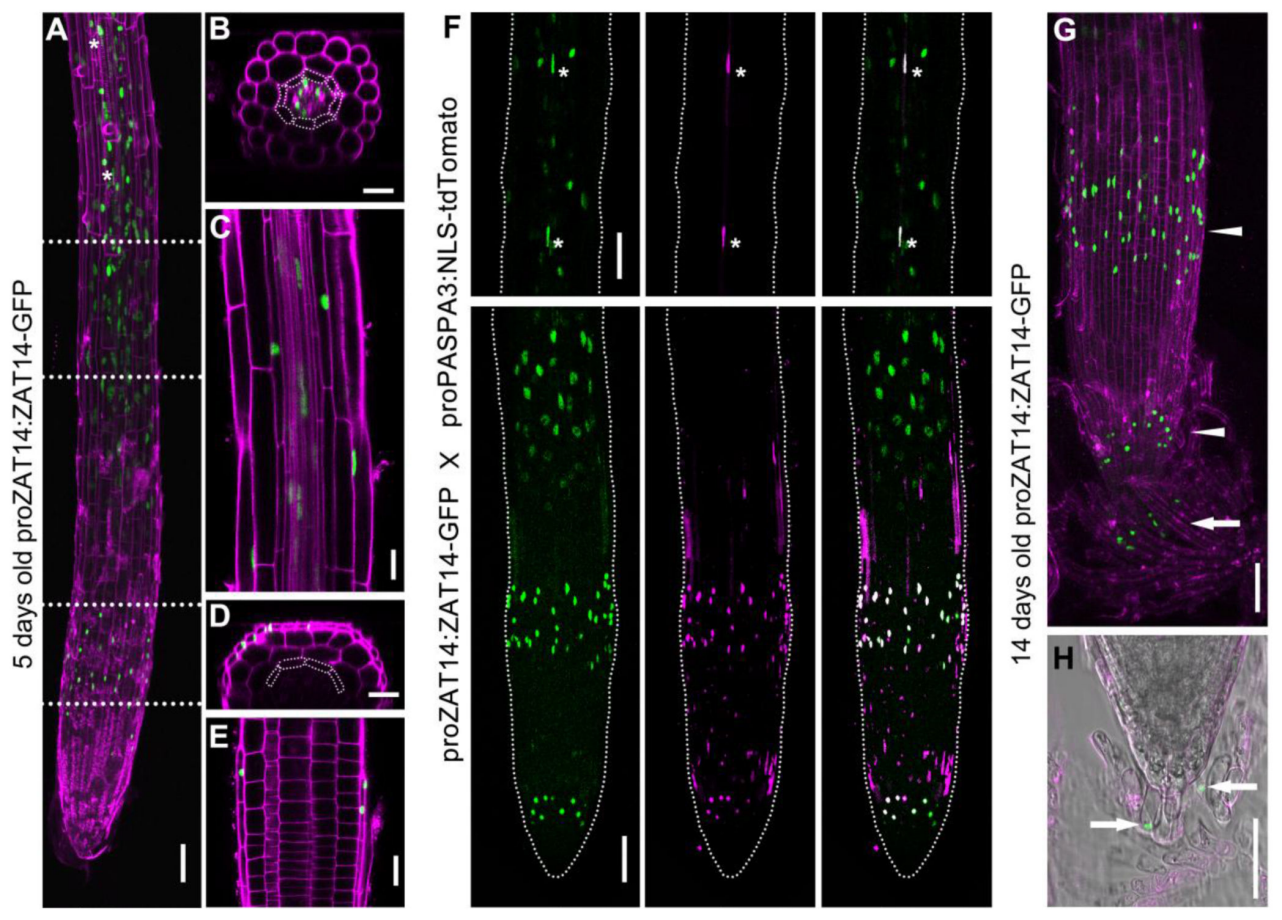
Expression patterns of ZAT14. (A-E) 5 days old root expressing *proZAT14:ZAT14-GFP*, stained with PI (in magenta). ZAT14-GFP was expressed in LRC prior to PCD and also showed signal in distal root (A). Cross-section (B) and longitudinal section (C) of distal root. Cross-section (D) and longitudinal section (E) of proximal root. Endodermis cells are marked by white dotted lines in (B) and (D). (A) consists of three stitched images. White dotted lines in (A) point at the imaging area for (B) and (C). (F) 5 days old root expressing *proZAT14:ZAT14-GFP* and *proPASPA3:NLS-tdTomato*. Asterisks indicate xylem cells expressing *ZAT14* and *PASPA3*. Distal root is shown as a single middle stack, while the proximal root is shown as a Z-projection. (G-H) 14 days old root expressing *proZAT14:ZAT14-GFP*, stained with PI. Projection section (G) and longitudinal section (H). The white arrowhead indicates distal and proximal LRC cells, and white arrows indicate columella cells. Bars = 50 µm for A, F,G, 20 µm for B to E, and H. See also Supplemental figure 2.

To visualize the gene expression pattern of *ZAT14*, as well as protein localization and dynamics, we generated transcriptional and translational reporter lines. In translationalreporterlines (*proZAT14:ZAT14-GFP*), 5-day-old seedlings showed strong nuclear-localized GFP signals in the LRC prior to PCD (Figure 3A, 3D, 3E), but alsoexpression in the root epidermis, endodermis and vasculature (Figure 3B, 3C). However, the expression of ZAT14 mRNA is highest in sc-RNAseq clusters of xylem and root cap tissues preparing for dPCD (Supplemental figure 2A). By crossing with the PCD-reporter line *proPASPA3:NLS-tdTomato*(Xuan et al., 2016),*proZAT14* was confirmed as co-expressingwith *proPASPA3* in the LRC and the xylem cells prior to PCD (Figure 3F), consistent with the scRNA-seq data. During the dynamic turnover of the root cap, PCDprogresses towards and into the columella(Fendrych et al., 2014; Huysmans et al., 2018), and also here we observed ZAT14-GFP expression (Figure 3G-H). The transcriptional reporter lines (*proZAT14:NLS-GFP-GUS*) generallyshowed a similar as the translational reporter line (Supplemental figure 2B). However, transcriptional reporters did not show expression in columella cells, suggesting the ZAT14 fusion protein might either be stabilized in, or transported to, the columella. GUS histochemical staining showed GUS signal in the root and leaf vasculature, at the junction of the anther filament as well as in the floral organ abscission zone (Supplemental figure 2C-G). Interestingly, GUS signals were also detected in the phloem of the inflorescence stem (Supplemental figure 2H), suggesting ZAT14 might fulfil functions in this cell type as well. These data suggest that *ZAT14* is expressed in tissues undergoing PCD, but not restricted to these tissues.

### *ZAT14* is downstream of the key root cap PCD regulator*SMB*

Considering that the *ZAT14* expression pattern is associated with cells undergoing dPCD in the root capand that *ZAT14* OE phenocopied the overexpression of SMB, we investigated therelationship between SMB and ZAT14 in the dPCD regulatory network.

First, we generated an RNA-seq dataset of a dexamethasone-inducible SMB-GR line (Bennett et al., 2010)6 hours after dexamethasone treatment (Supplemental dataset 2). Our analyses revealed that *ZAT14* and seven other members of the C1-2i subclass (*ZAT5*, *ZAT15*, *ZAT13*, *AT4G16610*, *ZAT14L*, *AZF3*, *AZF1*) showed at least 2-fold upregulation upon inducible SMB expression. Among these TFs, *ZAT5* and *ZAT14*were highly expressed in the root cap and upregulated during dPCD. They both show an increased expression close to 16-fold change, upon SMB overexpression (Supplemental table 1). These results suggestthat the expression of ZAT14 and related members is positively regulated by SMB.

**Figure 4:**
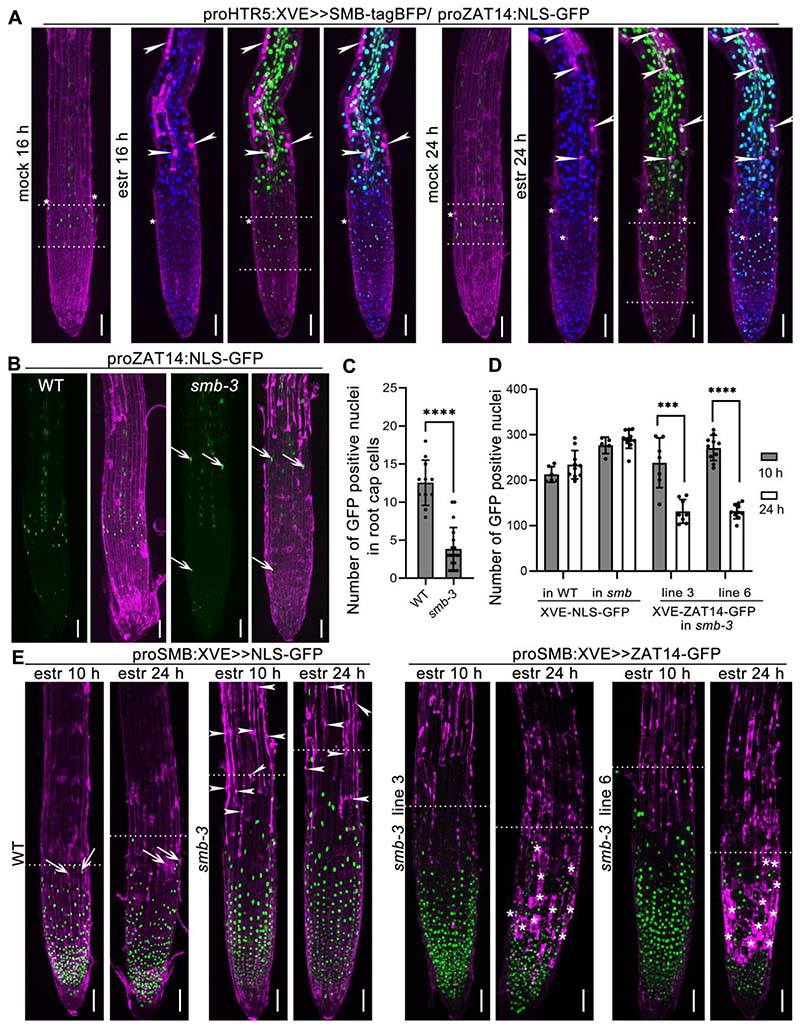
ZAT14 is downstream of SMB TF. (A) *proHTR5:XVE>>SMB-tagBFP* (in blue) was transformed in the reporter line *proZAT14:NLS-GFP-GUS* (in green) and stained with PI (in magenta), which have been treated with estradiol or DMSO (as mock) for 16 h or 24 h. Each panel consists of two stitched images. White asterisks indicate LRC PCD. White arrowheads indicate ectopic cell death. White dotted lines indicate size of LRC expressing *proZAT14:NLS-GFP-GUS*. Bars = 50 µm. (B) The ZAT14 transcriptional reporter line (in green) in WT and *smb-3* mutant background and stained with PI (in magenta). The white arrows point at the expression of *proZAT14* in *smb-3* mutant at the LRC cells. Each panel consists of three stitched images. Bars = 50 µm. (C) Quantification of GFP-positive LRC cells of ZAT14 transcriptional reporter line in WT and *smb-3* mutant was shown as means ± SD (n = 11 for WT, n = 19 for *smb-3*). Statistical analysis was performed by *t* test; ****P < 0.0001. (D) Quantification of GFP-positive nuclei in WT and *smb-3* inducibly expressing NLS-GFP, and two independent lines inducibly expressing ZAT14-GFP in *smb-3* mutant at 10 and 24 hours after induction. Results are means ± SD (n = 6, 10, 5, 11, 7, 8, 10, 10 for the columns from left to right). Statistical analysis was performed by *t* test; *** p < 0.001, ****P < 0.0001. (E) The *proSMB:XVE>>NLS-GFP*and *proSMB:XVE>>ANAC046-GFP* lines stained with PI (in magenta). *smb-3* mutant showed aberrant dPCD marked by PI. Induced expression of ZAT14-GFP under SMB promoter in the *smb-3* mutant background resulted in massive PI entry the cell 24 hours after estradiol treatment. The roots were imaged 10 and 24 h after induction with estradiol (estr). Each panel consists of two or three stitched images. The root cap size is indicated by white dotted lines. White arrowheads indicate cell corpses in *smb* mutant. White arrows point at LRC cell death in wild type. White asterisks point at increased cell death in root cap cells. Bars = 50 µm.

Second, when a SMB-TagBFP fusion protein was inducibly expressed under the control of the ubiquitous*HTR5* promoter and transformed into the transcriptional reporter line of ZAT14, we observed strong ectopic activation of *ZAT14*expression in cells that misexpressed SMB (Figure 4A).

Finally, we crossed the *smb-3* mutant(Willemsen et al., 2008)(hereafter called *smb*) with the transcriptional reporter line of ZAT14. We observed that reporterexpression in the root capwas weakened and occurred in less cells in *smb* mutant compared to wildtype with the reporter line (Figure 4B, 4C). This suggests that SMB contributes to ZAT14 expression in the root cap cells preparing for PCD, though ZAT14 expression does not seem to depend entirely on SMB activity.

Additionally, we investigated if a functional ZAT14-GFP fusion protein, expressed under the SMB promoter in the *smb* mutant, could complement the *smb*phenotype. The *smb*phenotype shows a longer root cap due to delayed dPCD, and an absence of corpse clearance after cell death (Fendrych et al., 2014) (Figure 4E, left panels).At 10 HAI in the*smb* background *proSMB:XVE>>ZAT14-GFP*was expressed throughout the root cap. At 24HAI, we observed increased cell death in the root cap, though this cell death was not restricted to the edge of the root cap as in the wild type (Figure 4E, left panels), but occurred in a more widespread fashion (Figure 4E, right panels). These results indicate that ZAT14-induced cell death can occur even in absence of SMB (Figure 4D, 4E). Altogether these results suggest that ZAT14 is downstream of SMB and does not require additional SMB-dependent genes to trigger cell death in the root cap.

### ZAT14 shares partially common PCD pathway with SMB

**Figure 5:**
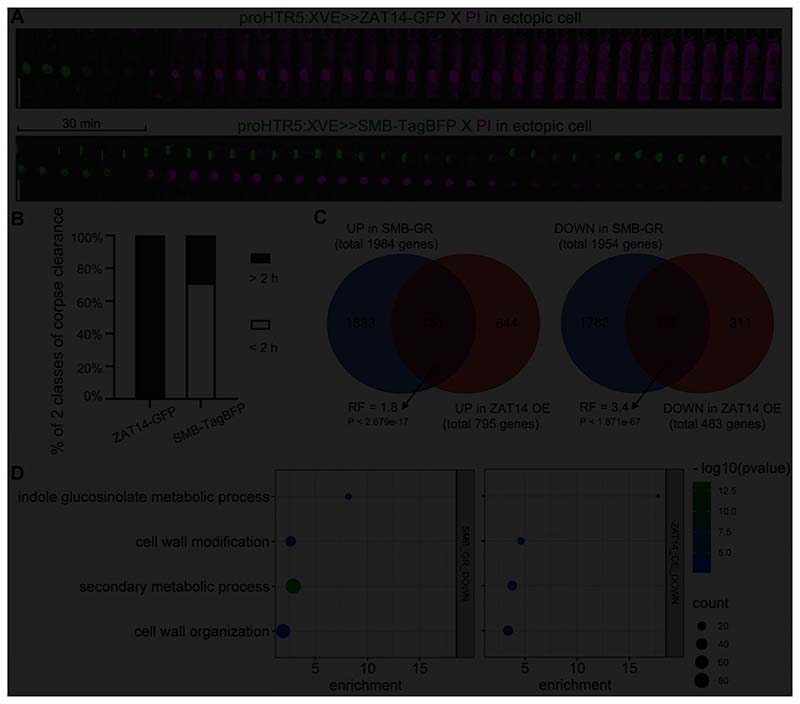
ZAT14 shares partially common PCD pathway with SMB. (A) Kymograph showed the corpse clearance in the epidermis in the inducibly overexpressing ZAT14-GFP (in green; upper panel) or SMB-TagBFP (in green; lower panel), stained with PI (in magenta). Cells were imaged every 5 min. Bars = 20 µm. (B) Quantitative analysis of corpse clearance. Percentage of cells showing cell corpses persisted without any signs of rapid autolysis is indicated in grey columns, whereas white columns indicate the percentage of cells showing corpse clearance within 2 hours. In total, 28 cells were analyzed for SMB-TagBFP and 18 cells for ZAT14-GFP. (C) Venn diagram of the upregulated genes and downregulated genes upon overexpression of ZAT14 and SMB. Differentially expressed genes with at least a 2-fold increased expression in the 6 hours dexamethasone-induced *pro35S:SMB-GR* compared to the mock (log_2_FC > 1, P < 0.05 and FDR < 0.05) -in total 1984 genes- were overlapped with differentially expressed genes that had at least a 2-fold increased expression in the 8 hours after estradiol induction *proG10-90:XVE>>ZAT14* (TPT lines) compared to the mock (log_2_FC > 1, P < 0.05 and FDR < 0.05) -in total 795 genes. This resulted in 151 genes. Differentially expressed genes with at least a 2-fold decreased expression in the 6 hours dexamethasone-induced *pro35S:SMB-GR* compared to the mock (log_2_FC < -1, P < 0.05 and FDR < 0.05) -in total 1954 genes- were overlapped with differentially expressed genes that had at least a 2-fold decreased expression in the 8 hours after estradiol induction *proG10-90:XVE>>ZAT14* compared to the mock (log_2_FC < -1, P < 0.05 and FDR < 0.05) -in total 483 genes. This resulted in 172 genes. RF is the representation factor meaning that the number of common genes is 1.8 and 3.4 times higher than what would be expected from two random lists of genes, for upregulated and downregulated common genes, respectively. (D) GO terms representing enriched biological processes derived from genes exclusively downregulated by overexpression of SMB (SMB-GR) and ZAT14 (ZAT14-OE). The most specific term from each family term provided by PLAZA was plotted along with the corresponding gene count (dot size), enrichment (x-axis), and p-value (colour scale; Bonferroni correction for multiple testing) represented as log_10_. Only common GO terms with a p-value below 0.05 in SMB-GR and ZAT14-OE were plotted. See also Supplemental figure 3.

Upon systemic inducible misexpression of *SMB*, *ANAC087*, and *ANAC046*, dPCD-associated genes are upregulated, indicative of an ectopically activated dPCD program (Fendrych et al., 2014; Huysmans et al., 2018). To investigate if ZAT14 indirectly promotes the expression of dPCD-associated genes as part of a core dPCD program, we tested whether the dPCD-associated genes *BFN1*, *EXI1*, *PASPA3* and *RNS3*are upregulated upon inducible misexpression of ZAT14. The qPCR analysis showed that none of the interrogated genes were upregulated (Supplemental figure 3A), suggesting that ZAT14 does not act upstream of the known dPCD-associated genes. In line with this hypothesis, ZAT14-conferred cell death was not followed by post-mortem corpse clearance, while SMB-triggered cell death was (Figure 5A-B). This suggests that cell clearance enzymes including BFN1 are not activated downstream of ZAT14 (Supplemental figure 3A).

To investigate the function of ZAT14, we performed a transcriptome analysis of an inducible ZAT14 OE line from the TPT collection at 8h after estradiol treatment versus a mock-treated control (Supplemental dataset 3). 795 genes were upregulated by ZAT14 OE, while 483 genes were downregulated compared to the mock-treated control (Figure 5C, Supplemental dataset 3). Confirming our qPCR results, PCD-associated genes including *PASPA3*,*BFN1*, *RNS3*, *MC9* and *DMP4*were not upregulated in ZAT14 OE (Supplemental dataset 3). To investigate a potential gene regulatory overlap between SMB and ZAT14, we compared up- and downregulated genesupon overexpression of *ZAT14* and *SMB*. 151 genes were commonly upregulated and 172 genes were commonly downregulated following ZAT14 and SMB OE. Both gene groups, but especially downregulated genes are much more abundant than expected by chance, as indicated by a representation factor of 3.4(Figure 5C).By contrast, not more genes than expected by chance were oppositely regulated by SMB OE and ZAT14 OE (Supplemental figure 3B,C). These results suggest that there is a common group of downregulated genes downstream of SMB and ZAT14. Commonly downregulated genes were enriched in GO terms including indole glucosinolate metabolic process, secondary metabolic process, cell wall modification and organization (Figure 5D). While no PCD-related GO-terms appeared enriched in commonly upregulated genes, several upregulated genes have been associated with cell death and senescence events, including*CYSTEINE ENDOPEPTIDASE 2* (*CEP2*, AT3G48340),*TATD RELATED DNASE* (*TATD*, AT3G03500) and*RESPONSIVE TO DEHYDRATION 21B*(*RD21B*, AT5G43060)(Supplemental dataset 4)(Shindo et al., 2012; Lampl et al., 2013; Howing et al., 2018; Buono et al., 2019).Our results indicate that ZAT14 might control a subset of the gene regulatory network downstream of SMB.As GO enrichment analysis did not identify putative mechanisms activated by ZAT14 misexpression, it remains a future challenge to understand ZAT14 function in the context of dPCD.

### ZAT14 and its homologs control the onset of PCD in columella cells

**Figure 6:**
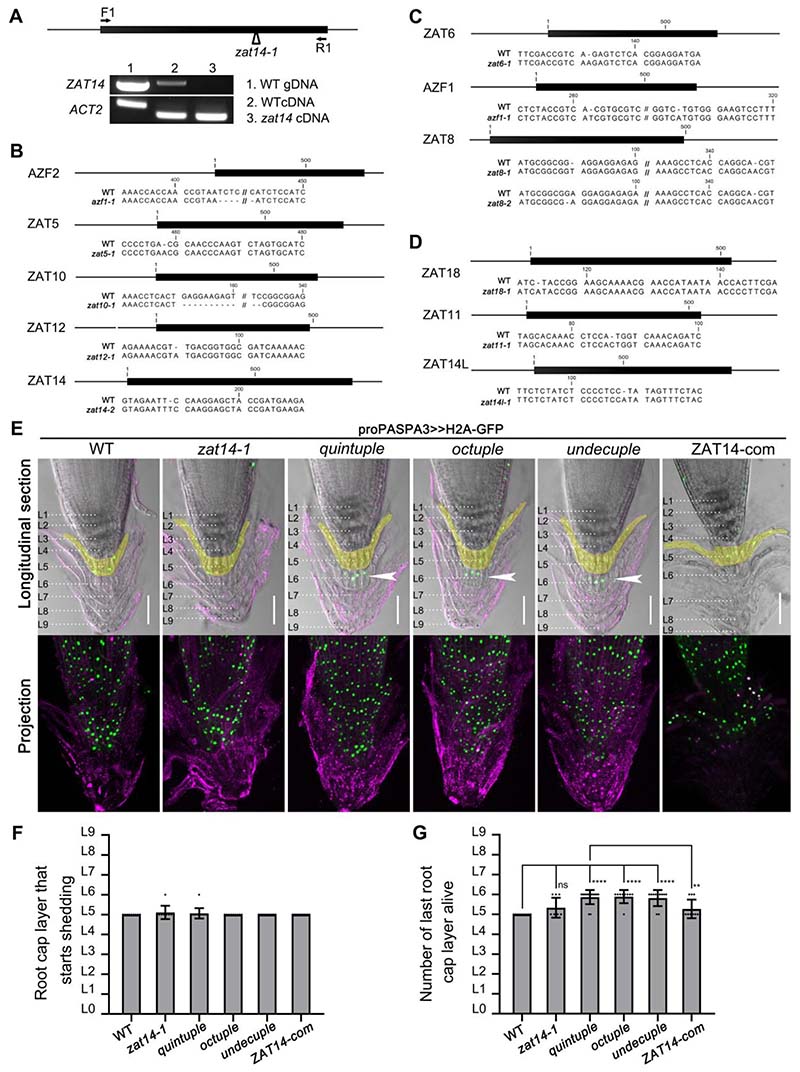
ZAT14 and its homologs control the onset of PCD in columella cells. (A) Schematic illustration of T-DNA insertion within the genomic region of *ZAT14* and transcript analysis of *ZAT14* in *zat14-1* mutant. Arrowhead points at the T-DNA insertion site. Arrows indicate the binding sites for RT-PCR primers. *ACTIN2 (ACT2)* was used as internal control for the RT-PCR. (B-D) Schematic illustration of genomic regions and CRISPR mutation sites of 11 C1-2i encoding genes, *AZF2*, *ZAT5*, *ZAT10*, *ZAT12*, *ZAT14*, *ZAT6*, *AZF1*, *ZAT8*, *ZAT18*, *ZAT11* and *ZAT14L*. (E) From left to right: representative root tips from *proPASPA3:H2A>>GFP* in the wild type, *zat14-1*, *ZAT* higher order mutants (stained with PI, in magenta) and quintuple mutant with *proZAT14:ZAT14-tdTomato* (ZAT14-com). *zat quintuple*: *azf2-1 zat5-1 zat10-1 zat12-1 zat14-2*; *zat octuple*: *azf2-1 zat5-1 zat10-1 zat12-1 zat14-2 azf1-1 zat6-1 zat8-1; zat undecuple*: *azf2-1 zat5-1 zat10-1 zat12-1 zat14-2 azf1-1 zat6-1 zat8-2 zat11-1 zat18-1 zat14l-1.* Columella root cap layers are marked as L1-L9. The fifth layer from each genotype is highlighted in light yellow. White arrows indicate there are more living columella layers in *zat* higher order mutants compared with wild type. Bars = 50 µm. (F) The quantification of the root cap layer where shedding starts. Results are means ± SD (more than 9 roots were quantified for each genotype). (G) The quantification of the living root cap layers for each genotype. Results are means ± SD (more than 9 roots were quantified for each genotype). Statistical analysis was performed by *t* test; ** p < 0.01, ****P < 0.0001, ns: not significant. See also Supplemental figures 4 and 5.

To investigate the role of ZAT14 in root cap PCD, we identified a mutant carrying a T-DNA insertion in the single exon of *ZAT14* (SALK_114288C; *zat14-1*) that was unable to generate a full-length transcript (Figure 6A).However, we did not find any obvious root cap PCD phenotype using a *proPASPA3>>H2A-GFP* dPCD reporter construct (Olvera-Carrillo et al., 2015)and fluorescein diacetate (FDA) combined with PI as a live-death stain(Huysmans et al., 2018) (Figure 6E, Supplemental figure 5C). To investigate whether genetic redundancy was responsible for the lack of *zat14-1* mutant phenotype, we generated higherorder mutants by multiplex CRISPR-Cas9 (Figure 6B). First, we targeted C1-2i subfamily members that are most highly expressed in the root cap prior to root cap dPCD: *ZAT14*, *AZF2*, *ZAT5*, *ZAT10* and *ZAT12* (Supplemental table 2). In a *proPASPA3>>H2A-GFP* reporter background, we isolated a *zatquintuple* mutant, confirming homozygous frameshift mutations leading to early stop codons in each gene in the T3 generation (Figure 6B and Supplemental figure 4). For most genes the stop codon was located upstream of the zinc finger motifs responsible for DNA binding, suggesting the alleles generate null mutants. An exception was AZF2 in which the stop codon occurred only after the first zinc finger motif. However, a truncated AZF2_Δ165aa-173aa_ construct was unable to cause cell death after transient expression in *N. benthamiana*, while the full-length AZF2 produced cell death (Supplemental figure 5A-B), suggesting that the isolated *azf2* allele is a null mutant.

In the LRC of 5-day-old quintuple mutant seedlings, we could not identify an aberrant *proPASPA3>>H2A-GFP* expression pattern, nor any obvious differences with the wild-type as visualized by PI staining (Supplemental figure 5C-D). However, when we cultivated seedlings for 14 days on vertical agar plates to allow the production of additional root cap layers(Feng et al., 2022), we observed a clear delay of dPCD in the columella root cap in the quintuple mutant compared to the wild-type and to the *zat14-1* mutant. In the quintuple mutant living cells were still detected in the sixth layer, while in the wild type and the *zat14-1* mutant these cells had already undergone PCD (Figure 6E, Supplemental figure 5E). The number of root cap layers and the columella shedding process were not different in the quintuple mutant (Figure 6F, G), suggesting that loss of ZAT proteins specifically compromised dPCD execution and not root cap development or maturation. We observed a partial reversion of this phenotype by expressing a *proZAT14:ZAT14-tdTomato* complementation construct (Figure 6E, G), demonstrating that ZAT14 plays a prominent role among redundantly acting ZAT proteins.

However, even in the quintuple mutant, we did not observe a dPCD phenotype in the distal LRC. To remove further redundancy, we generated an octuple and an undecuple ZAT mutant by targeting *ZAT8*, *ZAT6*, *ZAT14L*, *ZAT11*, *ZAT18* and *AZF1* in the *zat* quintuple mutant background (Figure 6C-E, Supplemental figure 5C-E). However, no distal LRC dPCD phenotype was observed in these mutants (Supplemental figure 5C-D), and the delayed columella PCD process was not exacerbated (Figure 6E).Possibly, there is more extensive redundancy within the C1-2i subfamily, or even beyond among repressive C2H2-type zinc finger proteins.

## Discussion

We have identified ZAT14 as a novel transcriptional regulator in the root cap dPCD gene regulatory network downstream of the established transcription factor SMB. In contrast to SMB and other NAC transcription factors involved in cell death control, ZAT14 contains a repressive EAR-motif (Englbrecht et al., 2004; Ciftci-Yilmaz and Mittler, 2008; Xie et al., 2019)that is conserved in the members of the C1-2i subfamily. We showed that deletion or mutation of the EAR-motif does not interfere with ZAT14’s capacity to cause cell death upon inducible misexpression.This finding stands in contrast to results showing that removal of the C-terminal EAR-motifs were sufficient to disturb the functions of ZAT10 and ZAT11 upon transient misexpression (Ohta et al., 2001). However, other approaches showed that growth-inhibition caused by ZAT7 overexpression did not depend on the presence of the EAR-motif (Ciftci-Yilmaz et al., 2007). Likewise, the osmotic stress phenotype of *zat10*mutants could be complemented by a ZAT10 protein with a mutated EAR motif (Nguyen et al., 2016).

Our results showed thatsimultaneous mutations in both L-box and EAR-motif were necessary to impair ZAT14 function, suggesting that these domains have redundant or complementary functions for ZAT14’s cell death regulatory capacity. The L-box in ZAT14 resembles the LXLXL motif in ZAT1 that functions as a transcription repression motif (Kagale and Rozwadowski, 2011; Song et al., 2020). The fact that we can reconstitute ZAT14 function by fusing a C-terminal SRDX domain to the ZAT14_mLmEAR_ version lacking both L-box and EAR motif suggests that ZAT14 indeed acts as a transcriptional repressor and that it needs both L-box and EAR-motif to exert this function. Together, these data suggest that repressive C1-2i ZAT proteins act as transcription repressors via the canonical EAR motif and other less well-defined repressive domains.

We identified ZAT14 as a dPCD-associatedgene that is co-expressed with established dPCD-associated genes in the root cap and protoxylem(Olvera-Carrillo et al., 2015).Like other dPCD-associated transcription factors, for instance,ANAC046 and ANAC087 (Huysmans et al., 2018), or VND6 and VND7 (Yamaguchi et al., 2010), inducible ZAT14 misexpression causes a rapid and widespread cell death, ultimately leading to the death of the entire plant. Interestingly, ZAT14-induced cell death occurs later in the root cap than in the root elongation zone. This might hint at the existence of root-cap expressed factors that specifically attenuate ZAT14 activity in this tissue.

We demonstrate that ZAT14 is downstream of SMB, the key regulator of root cap maturation and the LRC dPCD (Fendrych et al. 2014). The three NAC TFs involved in the LRC dPCD, SMB, ANAC046 and ANAC087 partially share PCD-associated genes as their targets (Huysmans et al., 2018). But these genes are not the targets of ZAT14 as shown by our RNA-seq analysis. This is not surprising given the fact that the established NAC TFs are considered to act as transcriptional activators, while ZAT14 is a transcriptional repressor. As a repressor, ZAT14 might suppress genes that inhibit the execution of the dPCD or post-mortem corpse clearance. Alternatively, ZAT14 function might suppress other pathways that attenuate cellular viability, and therefore indirectly contribute to dPCD promotion in the the columella root cap and the xylem.Further investigation of the downstream targets of ZAT14 are necessary to shed light on the involvement of ZAT14 in PCD promotion.

However, despite ZAT14 expression in both distal and proximal root cap dPCD zones we only found a loss of function phenotype of dPCD delay in the columella and adjacent proximal LRC cells in higher-order mutants. Possibly, there are additional ZAT-related transcription factorswithin or without the C1-2i subclass that act redundantly to ZAT14. Recently, ZAT1, ZAT4, and ZAT9 have been implicated in the maturation of root cap cells as positive regulators of the dPCD-associated gene *PASPA3*(Song et al., 2020). These genes belong to the C1-3i subclass of C2H2 zinc finger proteins. Even though according to a scRNA-seq dataset(Ferrari et al., 2022)ZAT1, ZAT4 and ZAT9 do not appear to be expressed in LRC cells prior to dPCD (Supplemental dataset 1), it is still possible that either these or other related genes are upregulated in the absence of highly expressed ZAT proteins in a compensatory fashion. This effect has been described for other gene families, though the underlying mechanism remains to be elucidated(El-Brolosy and Stainier, 2017). To further analyze the function of ZAT proteins in dPCD it might be necessary to generate higher-order mutants that knock out additional or alternative ZAT genes. However, such a task would be a risky endeavour given the number of potential additional genes to mutate and putative compensatory upregulation of lowly expressed paralogs.

An alternative possibility is that ZAT protein activity is dispensable for dPCD in the distal LRC, despite ZAT14 upregulation in this tissue. This situation is analogous to the fact that autophagy is activated in both the dying columella and distal LRC cells, but only required for timely PCD execution in the columella(Feng et al., 2022). Possibly, there are additional cell death mechanisms that operate in the distal LRC, but not in the columella, that can compensate for loss of ZAT14 function.

In conclusion, we have identified ZAT14 as a repressive transcription factor that operates in Arabidopsis root cap cell death downstream of the key regulator SMB. While ZAT14 misexpression is sufficient to cause ectopic cell death, knock out of *ZAT14* and up to 10 related root cap expressed paralogs show a delay of cell death execution in the columella cell type of the root cap. Future studies will have to reveal how the transcriptional repression of ZAT14 target genes contributes to preparing for and executing root cap PCD.

### Accession numbers

Sequence data from this article can be found in the Arabidopsis Genome Initiative or GenBank/EMBL databases under the following accession numbers: *AZF1* (AT5G67450), *AZF2* (AT3G19580), *BEARSKIN2* (*BRN2*, AT4G10350), *BFN1* (AT1G11190), *EXI1* (AT2G14095), *KIR1* (AT4G28530), *PASPA3* (AT4G04460), *RNS3* (AT1G26820), *SMB* (AT1G79580), *TMO5* (AT3G25710), *ZAT5* (AT2G28200), *ZAT7* (AT3G46090), *ZAT6* (AT5G04340), *ZAT8* (AT3G46080), *ZAT10* (AT1G27730), *ZAT11* (AT2G37430), *ZAT12* (AT5G59820), *ZAT14* (AT5G03510), *ZAT14L* (AT5G04390), *ZAT18* (AT3G53600).

## Materials and methods

### Plant materials, growth, and transformation

All *Arabidopsis thaliana* seedlings were grown vertically on 1/2 Murashige and Skoog (MS) medium (2.15 g/L MS salts, 0.1 g/L MES, pH 5.8 [KOH], and 0.8% plant agar) for 5 or 14 days in a continuous light room (intensity 120 µmol m^2^ s^-1^, 21°C) before analysis, unless stated otherwise. The Arabidopsis Columbia-0 (Col-0) ecotype was used as the wild type.The T-DNA insertion line *zat14-1* (SALK_114288C) was obtained from the Nottingham Arabidopsis Stock Centre. For verification of homozygous mutants, primers P1/P2 were used together with SALK specific left border primer P3. Primers were listed in Supplemental table 3. The following lines were previously described: the T-DNA insertion line *smb-3* (SALK_143526C)(Willemsen et al., 2008), the transgenic line *pro35S:SMB-GR*(Bennett et al., 2010), the marker line*proPASPA3>>H2A-GFP*(Olvera-Carrillo et al., 2015), *proPASPA3:NLS-tdTomato*(Xuan et al., 2016)and *proSMB:NLS-GFP*(Fendrych et al., 2014; Xuan et al., 2016). Transgenic plants were selected on 1/2 MS medium supplemented with 20 mg/mL Basta salts(Sigma-Aldrich) or based on the fluorescence color of seeds coat by stereo microscope.

### Pharmacological treatment

The stock solution of propidium iodide (Sigma-Aldrich, P4864-10 mL, 1 mg/mL in water) was diluted and added to 1/2 MS medium at the final concentration of 10 µg/mL.

The stock solution of Fluorescein diacetate (Sigma-Aldrich, F7378-5 g) was prepared using acetone as the solvent at the concentration of 2 g/mL. The stock solution was diluted and added to 1/2 MS medium at the final concentration of 2 µg/mL.

The stock solution of β-Estradiol (Sigma-Aldrich, E8875-1 g) anddexamethasone (Sigma-Aldrich, D4902-1 g) were prepared using DMSO as the solvent at the concentration of 50 mM/L. The β-Estradiol stock solution was diluted and added to 1/2 MS medium at the final concentration of 50 µM/L for spraying and 10 µM/L for plates. The dexamethasone stock solution was diluted and added to 1/2 MS medium at the final concentration of 10 µM/L. DMSO was equally diluted as the controls.

### TRANSPLANTA screening

To perform the screening of the TRANSPLANTA collection (Coego et al., 2014), seeds were germinated directly on 1/2 MS medium complemented with 10 µM β-Estradiol in 8.5cm diameter round plates. After sowing around 200 seeds and two days of vernalization at 4°C, the round plates were placed horizontally in a continuous lightroom (intensity 120 µmol m^2^ s^−1^) at 21°C. Pictures were taken 10 days after germination.

### Cloning

All fragments were cloned into Gateway-compatible entry clones (Invitrogen). To create the entry clones, DNA fragments were amplified using specific primers and high-fidelityDNA polymerase (BioLabs) in a standard PCR. All cloning primers are listed in Supplemental table 3.

The 1803 bp promoter fragment upstream of the start codon of ZAT14 was amplified from genomic DNA using primers P20/P21, and cloned into pDONRP4P1R using BP Clonase (Invitrogen) to obtain pEN-L4-proZAT14-R1. The ZAT14 coding sequences with and without stop codon were amplified from seedling cDNA using primers P22/P23 and P22/P24, respectively, and both PCR products were recombined into pDONR221 using BP Clonaseto obtain pEN-L1-ZAT14*-L2 and pEN-L1-ZAT14-L2.The ZAT14_∆C_ was subcloned from pEN-L1-ZAT14-L2 using primers P22/P25. The ZAT14_mEAR_ and ZAT14_∆L_ were subcloned from pEN-L1-ZAT14-L2 using primers, P22/P26/P27/P24 for ZAT14_mEAR_,P22/P28/P29/P24 for ZAT14_∆L_ by overlap PCR. The ZAT14_mLmEAR_ was subcloned from pEN-L1-ZAT14_mEAR_-L2 using primers P22/P28/P30/P24 by overlap PCR. The ZAT14_∆L∆C_was subcloned from pEN-L1-ZAT14_∆L_-L2 using primers P22/P25. The ZAT14_mLmEAR_SRDX was subcloned from pEN-L1-ZAT14_mLmEAR_-L2 using primers P22/P35. All fragments were recombined into pDONR221 using BP Clonase to obtain pEN-L1-ZAT14_∆C_-L2, pEN-L1-ZAT14_mEAR_-L2, pEN-L1-ZAT14_∆L_-L2, pEN-L1-ZAT14_∆L∆C_-L2, pEN-L1-ZAT14_mLmEAR_-L2 and pEN-L1-ZAT14_mLmEAR_SRDX-L2, respectively. The AZF2 coding sequence without stop codon was amplified from seedling cDNA using primers P31/P32. The AZF2_∆223-273aa_ and AZF2_∆165-273aa_ were subcloned from pEN-L1-AZF2-L2 using the primers, P31/P33 and P31/P34, respectively. PCR products were recombined into pDONR221 using BP Clonase to obtain pEN-L1-AZF2-L2, pEN-L1-AZF2_∆223-273aa_-L2, pEN-L1-AZF2_∆165-273aa_-L2. Entry vector pEN-R2-mTFP1*-L3 was published previously (Pasin et al., 2014). Other entry clones pEN-L1-NLS-GFP-L2, pEN-R2-GUS*-L3, pEN-R2-GFP*-L3, pEN-L1-SMB-L2, pEN-R2-TagBFP*-L3, pEN-L4-pro35S-R1, pEN-L4-proSMB:XVE-R1, pEN-L4-proHTR5:XVE-R1 and destination vectors pB7FWG2.0, pB7m34GW-FASTgreen and pB7m24GW-FASTgreen were obtained from the VIB-UGent plasmid repository (https://gatewayvectors.vib.be). The obtained entry clones were recombined into Gateway destination vector using LR Clonase II enzyme mix (Invitrogen) to create the expression clones. The expression vectors and its combination are listed in Supplemental table 4.

### CRISPR vectors and mutants

For the quintuple mutant, four guide RNAs (gRNAs) were designed for *AZF2*, *ZAT5*, *ZAT10*, *ZAT12*, and *ZAT14,* respectively. Cloning of gRNA vectors was performed as previously described (Decaestecker et al., 2019). Briefly, these gRNAs were annealed and ligated into their respective GG entry plasmid (pGG-A-AtU6-ccdB-B, pGG-B-AtU6-ccdB-C, pGG-C-AtU6-ccdB-D, pGG-D-AtU6-ccdB-E, pGG-E-AtU6-ccdB-F). Each entry clone targets one specific ZAT TF by one of the four designed gRNAs, but each position is the pool of four gRNAs of each gene. The gRNA entry plasmids were cloned into the pFASTGK_AtCas9_AG destination vector (VIB-UGent plasmid repository (https://gatewayvectors.vib.be)) using Golden Gate assembly. The obtained expression clones were transformed into *proPASPA3>>H2A-GFP* reporter line using the *Agrobacterium tumefaciens* floral dip method (Clough and Bent, 1998). 24 T1 plants were genotyped using Singer sequencing and one of these 24 T1 plants showed mutation for all 5 targeted genes, *AZF2*, *ZAT5*, *ZAT10*, *ZAT12*, and *ZAT14*. FAST negative T2 seeds (where the *Cas9* gene is absent) derived from this line were selected for genotyping to obtain homozygous lines for each gene. The quintuple mutant was obtained and confirmed in T3. Two gRNAs were designed for *ZAT6*, *AZF1*, *ZAT8*, *ZAT11*, *ZAT18* and *ZAT14L*, respectively. And these two gRNAs per each gene were ligated into their respective GG entry plasmid (pGG-A-AtU6-ccdB-B, pGG-B-AtU6-ccdB-C, pGG-C-AtU6-ccdB-D, pGG-D-AtU6-ccdB-E, pGG-E-AtU6-ccdB-F, pGG-F-AtU6-ccdB-G). Each entry clone targets one specific ZAT TF by two designed gRNAs. The gRNA entry plasmids were cloned into the pFASTGK_AtCas9_AG destination vector using Golden Gate assembly. The obtained expression clone was transformed into quintuple mutant to obtain octuple and undecuple mutants. All primers for cloning and genotyping were listed in Supplemental table 3.

### RT-PCR, RT-qPCR

Total RNA was isolated from 5 days after gemination seedlings of *zat14-1* mutant and wild type. Primers used in RT-PCR were P1/P2 for *ZAT14*, and P4/P5 for *ACTIN2*. For RT-qPCR of inducible overexpression lines, about 15 FAST positive seeds were selected from T2 seeds and sowed on 1/2 MS plates. 5 days old seedlings were treated with estradiol or mock for 8 h and then they were harvested. For each line, three biological replicates (independently repeated treatments) were tested. After RNA extraction using the Spectrum Plant Total RNA Kit (Sigma-Aldrich), 1 µg of RNA was used for DNA synthesis with the qScript cDNA SuperMix (Quantabio). The RT-qPCR was performed with the LightCycler 480 (Roche) using SYBR green for detection of double-stranded DNA. Analysis of the RT-qPCR data was done using https://intra.psb.ugent.be/qPCR/index.pl with *PEX4* (AT5G25760) and *UBL5* (AT5G42300) as housekeeping genes, and later the data were normalized against wild-type or mock samples. All primers are listed in Supplemental table 3.

### ZAT14 OE and SMB-GR RNA sequencing

TRANSPLANTA ZAT14 OE line and Col-0 whole seedlings, were treated either with DMSOor estradiol for 8 h. Then, they were harvestedas three biological replicates for each condition. The whole seedlings of SMB-GR line were treated with DMSO or DEX for 6 h before they were collected as three biological replicates.Each replicate contained 25 seedlings. The RNA was extracted as described above. The quantity and quality of the RNA was checked using a NanoDrop 2000 (Nanodrop Technologies Wilmington), and RNA integrity was confirmed on an Agilent 2100 Bioanalyzer (Agilent Technologies). The RNA-sequencing was performed by VIB Nucleomics core as Illumina NEXT-seq with 75 bp paired-end sequencing. Sequencing quality as well as read mapping and summarization were performed with a software pipeline on an in-house Galaxy server. Briefly, quality of raw data was verified with FastQC (https://www.bioinformatics.babraham.ac.uk/projects/fastqc/). Next, quality filtering was performed using Trimmomatic as described (Bolger et al., 2014). Reads were subsequently mapped to Arabidopsis genome (Araport11) (Cheng et al., 2017).

DEGs were identified withthe Edge-R software package in R (Robinson et al., 2010). Genes were considered as differentially expressed if their expression levels had an absolute Log_2_(FC) > 1, p < 0.05 and FDR < 0.05. For upregulated genes upon overexpression of ZAT14 (Supplemental dataset 3), firstly, we compared ZAT14 overexpressor line treated with estradiol (ZAT14_OE_Estr) with the same line after mock treatment (ZAT14_OE_mock), 1126 upregulated genes were obtained, while by comparing ZAT14_OE_Estr with estradiol-treated wild type (WT_Estr)there are 1055 upregulated genes. To remove the genes regulated by estradiol treatment or transgenic insertion, these two upregulated gene lists were combined and 795 genes were obtained and regarded as upregulated upon the overexpression of ZAT14. The same method was performed to get the downregulated genes, by comparing ZAT14_OE_Estr with ZAT14_OE_mock or ZAT14_OE_Estr with WT_Estr, 663 genes and 815 genes were obtained respectively. 483 downregulated genes were obtained after combing these two downregulated gene lists.

GO enrichment analyses were carried out with the PLAZA 5.0 website (https://bioinformatics.psb.ugent.be/plaza/versions/plaza_v5_dicots). GO pathway enrichment bubble plot was plotted by https://www.bioinformatics.com.cn/en a free online platform for data analysis and visualization.

For representation factor calculation, the online software: http://nemates.org/MA/progs/overlap_stats.html was used. "The number of genes expressed in the RNAseq" was considered as "Number of genes in the genome".

### Confocal imaging

Confocal images were acquired using LSM710 (Zeiss) and SP8X (Leica) microscopes. GFP and FDAwere excited by the 488nm line of the argon laser andwere detected between 500 and 550 nm, whereas PI was excited by the 561nm line of the laser and was detected between 580 and 740 nm. TagBFP was excited by the 405nm UV diode laser and detected between 425nm and 475nm. mTFP1 was excited by the 458nm line of the argon laser and detected between 495 and 550 nm.For time course, the seedlings of the *proHTR5:XVE>>ZAT14-GFP* and *proHTR5:XVE>>NLS-GFP* were grown for 5 d on 1/2 MS medium and then transferred to a Nunc Lab-Tek Chamber (Thermo Fisher) covered by an agar slab (1/2 MS containing 10 µM/L estradiol and 10 µg/mL PI). After transfer, the seedlings were returned to the growth room for 6 h and then imaged every 30 min for 20 h. Images were processed and analyzed using Fiji (https://fiji.sc/).

### GUS histochemistry

5-day-old seedlings expressing *proZAT14:NLS-GFP-GUS*wereincubated in X-Gluc staining buffer at 37°C in the dark overnight. Chlorophyll was removed by two sequential incubations in 70% ethanol for several hours at each step. After rehydration, samples were transferred to 50% glycerol for mounting on glass slides. Samples were photographed using an Olympus BX51 (Olympus)microscope with a 10× objective (numerical aperture 0.25) and LEICA IC90 E (Leica) stereo microscope.

### *Nicotiana benthamiana* infiltration

Agrobacterium strain LB4404 carrying the constructs of interest in infiltration media (10 mM MgCl_2_, 10mM MES at pH 5.6, 0.1mM acetosyringone) were infiltrated in the abaxial side of *N. benthamiana* leaves of 4-6 weeks plants. At least three representative infiltrated leaves were observed by confocal microscopy 2 days after infiltration. For cell death phenotype observations, pictures of *N. benthamiana* leaves were taken 7 days after infiltration.

### Data and statical analysis

Root lengths were measured in Fiji (https://fiji.sc/). For the statistical analysis,*t*-tests were calculated in GraphPad Prism (version 9.0.0, https://www.graphpad.com/). The Venn diagrams were created in the publicly available webtool (https://bioinformatics.psb.ugent.be/webtools/Venn/). The dotplot presenting the scRNA-seq data was prepared in RStudio (version 1.4.1103) using the packages ggplot2 and Seurat. The schemes for different protein versions and sequence alignments were modified and exported in CLC Main Workbench20.

## Supplementary Material

Dataset S1

Dataset S2

Dataset S3

Dataset S4

Figures S1-S5

Movie Legends

Movie S1

Movie S2

Table S1

Table S2

Table S3

Table S4
