# Supplementary material for: Repressive C2H2 zinc finger ZAT proteins promote programmed cell death in the Arabidopsis columella root cap
Source: Plant Physiol. Author manuscript; Available in PMC 2023 Nov 30. (PMC10231456; doi:10.1093/plphys/kiad130)
Supplement: Figures S1-S5 [file EMS171097-supplement-Figures_S1_S5.pdf]

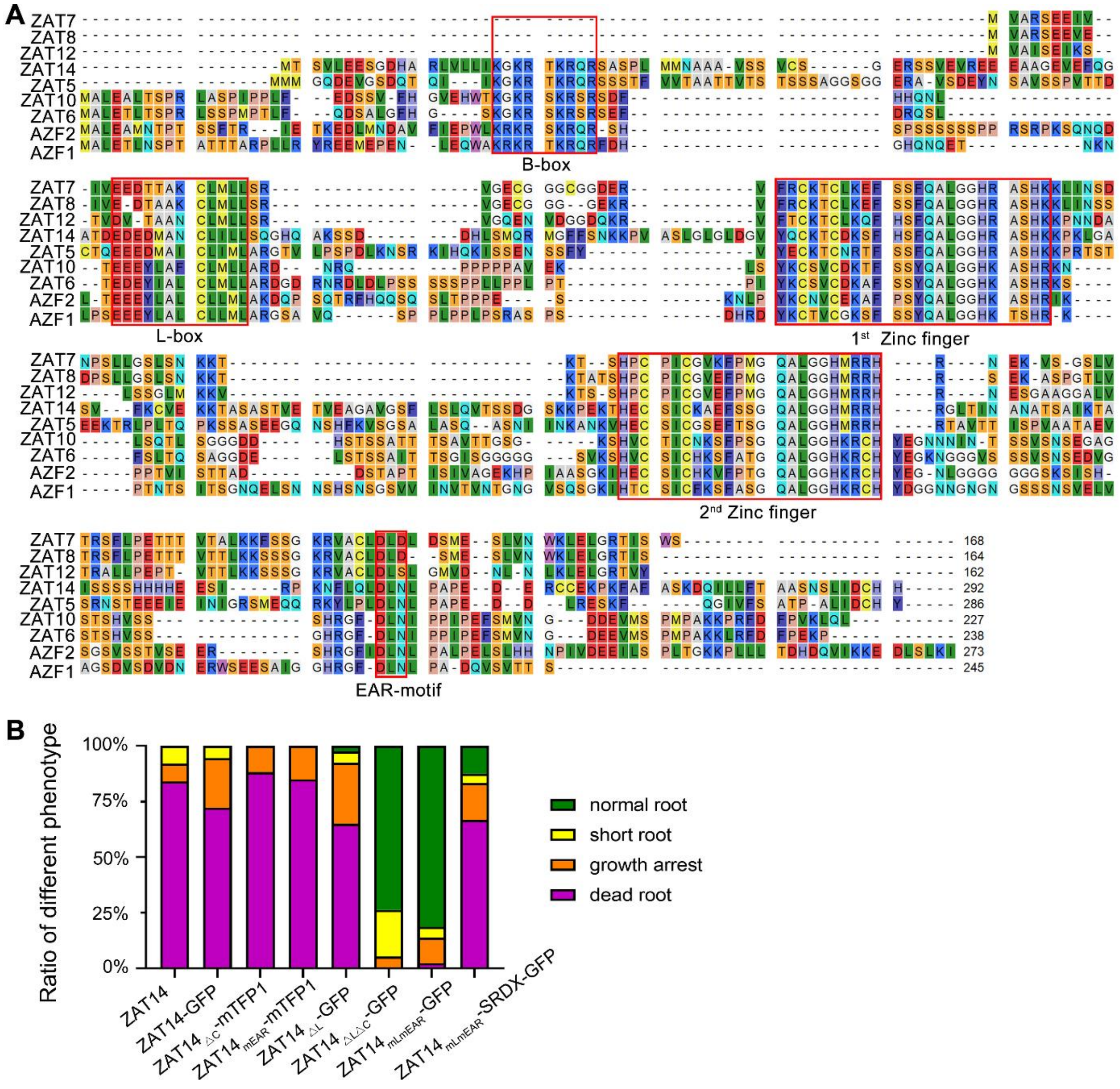

Supplemental figure 1. Sequence alignment and quantification of cell death phenotype.

(A) Multiple sequence alignment of ZAT14 and its close homologues. Conserved regions (B-box, L-box, zinc-finger domains and EAR-motif) are highlighted in red box.

(B) Quantitative analysis of cell death phenotype. Several independent T2 lines of each construct were treated with estradiol for 7 days for cell death phenotype analysis. At least 20 roots of each T2 line were analyzed. The number of lines for each construct: XVE-ZAT14 (n = 25), XVE-ZAT14-GFP (n = 20), XVE-ZAT14$_{\Delta C}$-GFP (n = 17), XVE-ZAT14$_{mEAR}$-GFP (n = 20), XVE-ZAT14$_{\Delta L}$-GFP (n = 40), XVE-ZAT14$_{\Delta L\Delta C}$-GFP (n = 38), XVE-ZAT14$_{mLmEAR}$-GFP (n = 29), XVE-ZAT14$_{mLmEAR}$SRDX-GFP (n = 24). Phenotypes were classified as: dead root (magenta), growth arrest (orange), short root (yellow), and normal root (green) when the root growth was unaffected by the treatment.

Related to Figure 2.

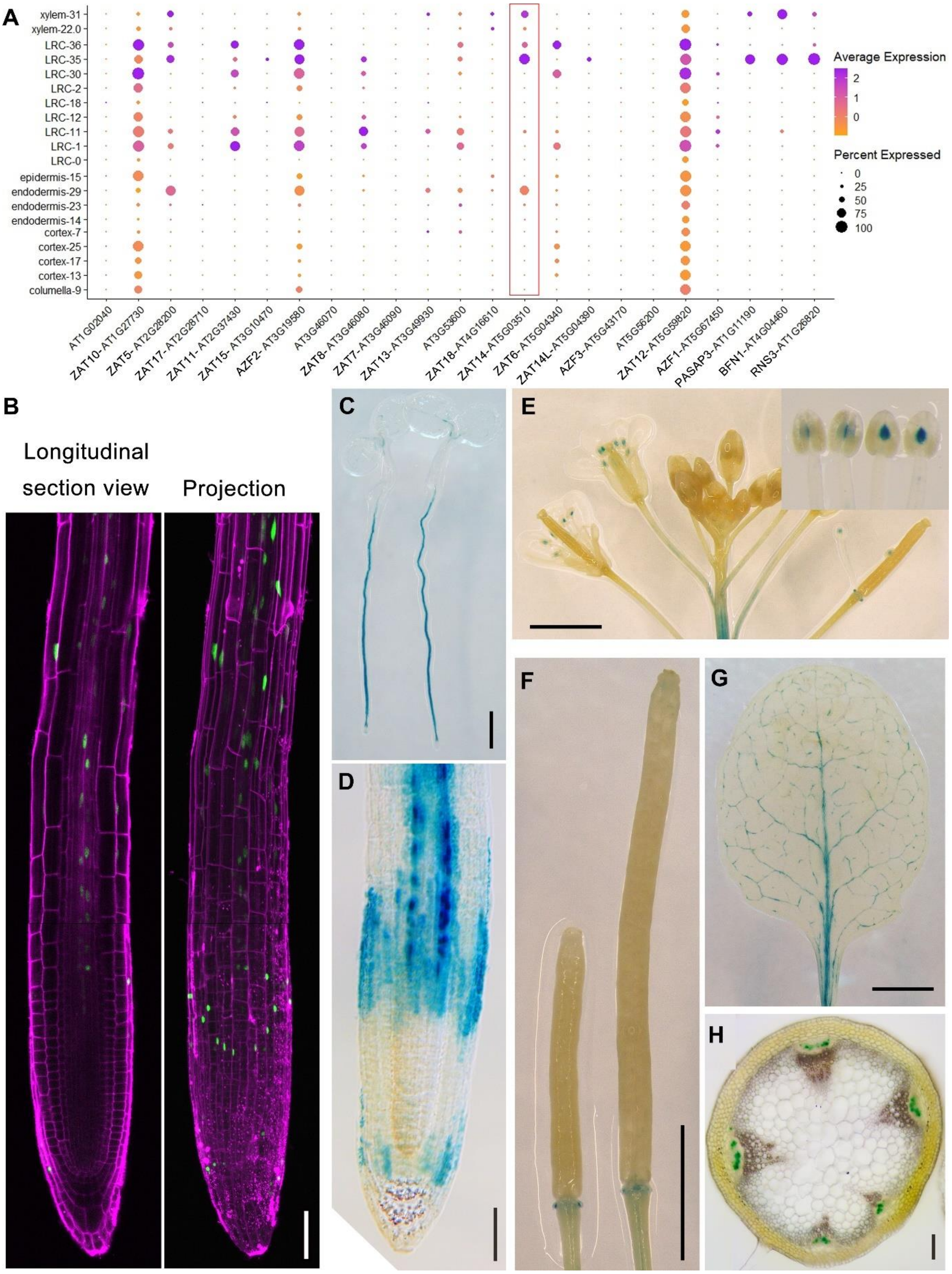

Supplemental figure 2. Expression patterns of ZAT14.

(A) Dotplot shows the expression pattern of 20 C2H2 protein family members in columella, LRC, xylem, cortex, epidermis and endodermis clusters obtained from root tip single cell RNA sequencing. High expression of PCD-associated genes (PASPA3, BFN1, RNS3) was noted in LRC

cluster 35 and xylem cluster 31 suggesting that these clusters belong to cells preparing or undergoing the dPCD. The red box marks expression pattern of ZAT14. Size of the dots corresponds to the percentage of cells expressing the gene within the cluster, the color reflects the expression levels (yellow is minimum, dark purple is maximum).

(B) The *proZAT14:NLS-GFP-GUS* reporter was expressed in the LRC cells undergoing dPCD, and in other root tissues in the upper part of the root. Each panel consists of two stitched images.

(C-H) GUS assay for the *proZAT14:NLS-GFP-GUS* line shows the expression of ZAT14 in other Arabidopsis organs. GUS signal was observed in the root vasculature of the Arabidopsis seedling (C, D), in the leaf vasculature (G), at the junction of the anther filament (E) as well as in the floral organ abscission zone (F), and in phloem cells in 4 weeks old stem (H). Bars = 50 µm for A and C, 0.5 cm for B, D to F, 100 µm for G.

Related to Figure 3.

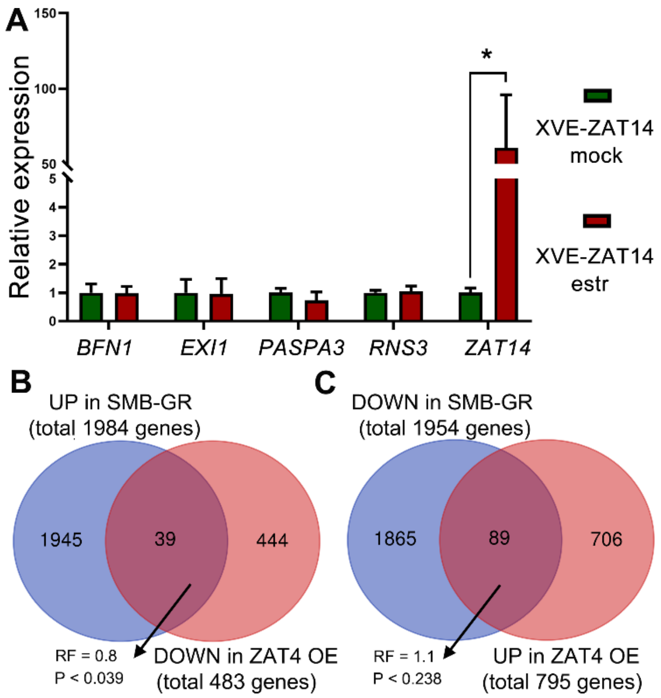

Supplemental figure 3. ZAT14 does not regulate the expression of several canonical dPCD-associated genes.

(A) *BFN1*, *EXI1*, *PASPA3* and *RNS3* dPCD markers are not significantly upregulated upon ZAT14 overexpression 8 h after estradiol (estr) treatment, as indicated by qPCR. Results shown are means ± SD (three independent treatments and three technical repeats each). Statistical analysis: *t* test; * p<0.05.

(B) Differentially expressed genes (DEG) which were at least 2-fold upregulated in the *pro35S:SMB-GR* after the 6 h treatment with dexamethasone (DEX) compared to mock-treatment ($log_2FC > 1$), in total 1984 genes, were overlapped with at least 2-fold downregulated genes in the 8 h estradiol-treated *proG10-90:XVE>>ZAT14* compared to the same mock-treated line ($log_2FC < -1$), in total 483 genes. This resulted in 39 genes. Representation factor = 0.8.

(C) DEGs which were at least a 2-fold decreased in the *pro35S:SMB-GR* line after 6 h of dexamethasone treatment compared to the same mock-treated line ($log_2FC < -1$), in total 1954 genes, were overlapped with DEGs with at least a 2-fold upregulation in the 8 h estradiol treatment of *proG10-90:XVE>>ZAT14* compared to the same mock-treated line ($log_2FC > 1$), in total 795 genes. This resulted in 89 genes. Representation factor = 1.1.

Related to Figure 5.

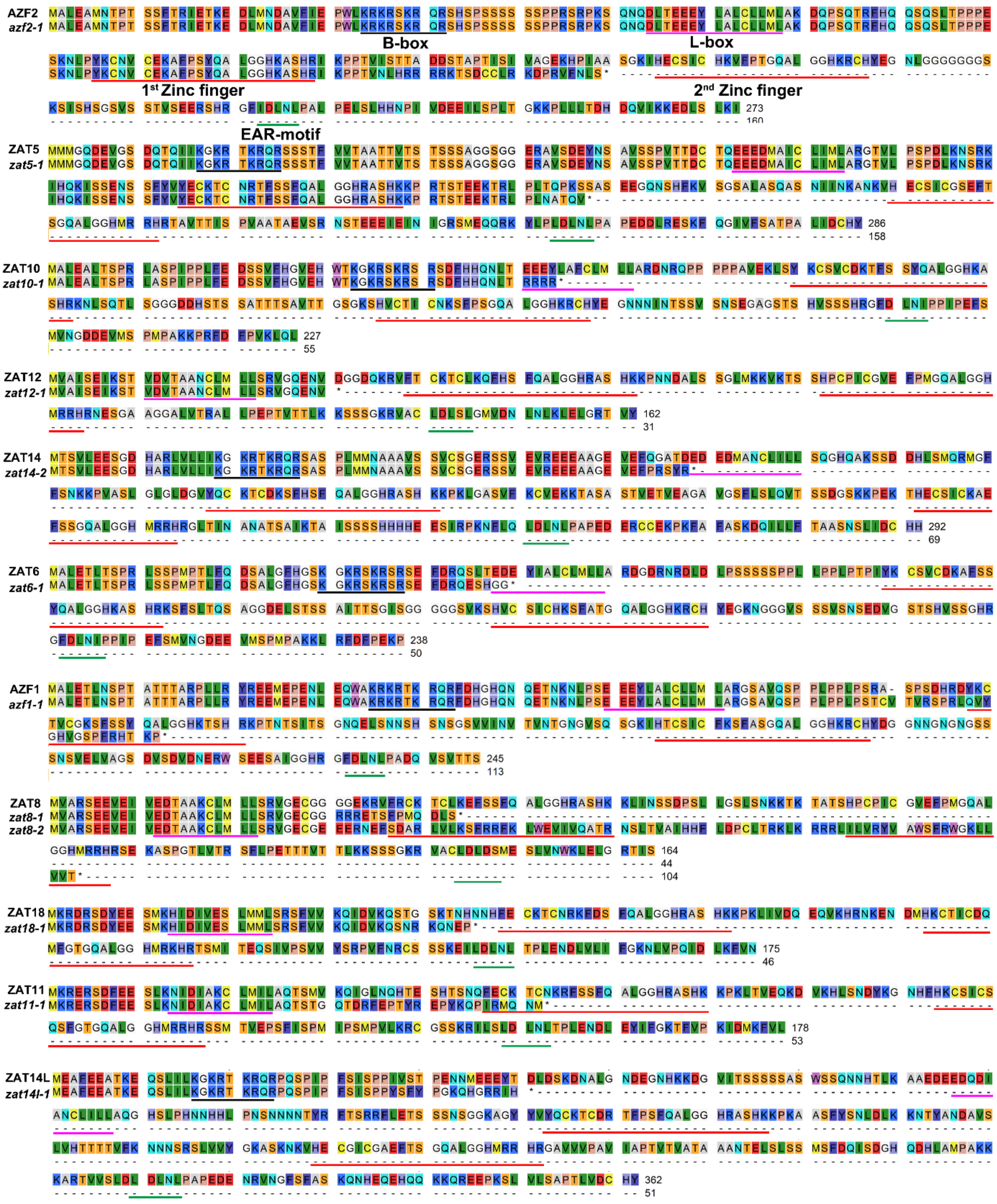

Supplemental figure 4. Amino acid sequence alignment of the targeted genes in *zat* mutants, *AZF2, ZAT5, ZAT10, ZAT12, ZAT14, ZAT6, AZF1, ZAT8, ZAT18, ZAT11* and *ZAT14L*, showed disrupted protein sequence. All mutations resulted in frameshift. Black lines mark B-box. Magenta lines mark L-box. Red lines mark zinc finger domain. Green lines mark EAR-motif. In *azf2-1*, there has been a 35 bp deletion (407bp-441bp) causing a stop codon 160 aa before the second zinc finger domain. In *zat10-1*, there is a 182 bp deletion (151bp-332bp), leading to a stop codon at 55 aa before the L-box. In *zat5-1*, *zat12-1*, *zat14-2, zat6-1*, *azf1-1*, *zat8-1*,

zat18-1, *zat11-1*, *and zat14l-1*, there is a base pair insertion leading to an early stop codon: at 158 aa after the first zinc finger domain in *zat5-1*, at 31 aa after the L-box in *zat12-1*, at 69 aa before the L-box in *zat14-1*, at 50 aa before the first zinc finger domain in *zat6-1*, at 113 aa during the first zinc finger domain in *azf1-1*, at 44 aa within the L-box in *zat8-1*, at 46 aa before L-box in *zat18-1*, at 53 aa within the L-box in *zat11-1* and at 51 aa before L-box in *zat14l-1*. In *zat8-2*, there is a base pair deletion causing a stop codon at 104 aa during the second zinc finger domain.

Related to Figure 6.

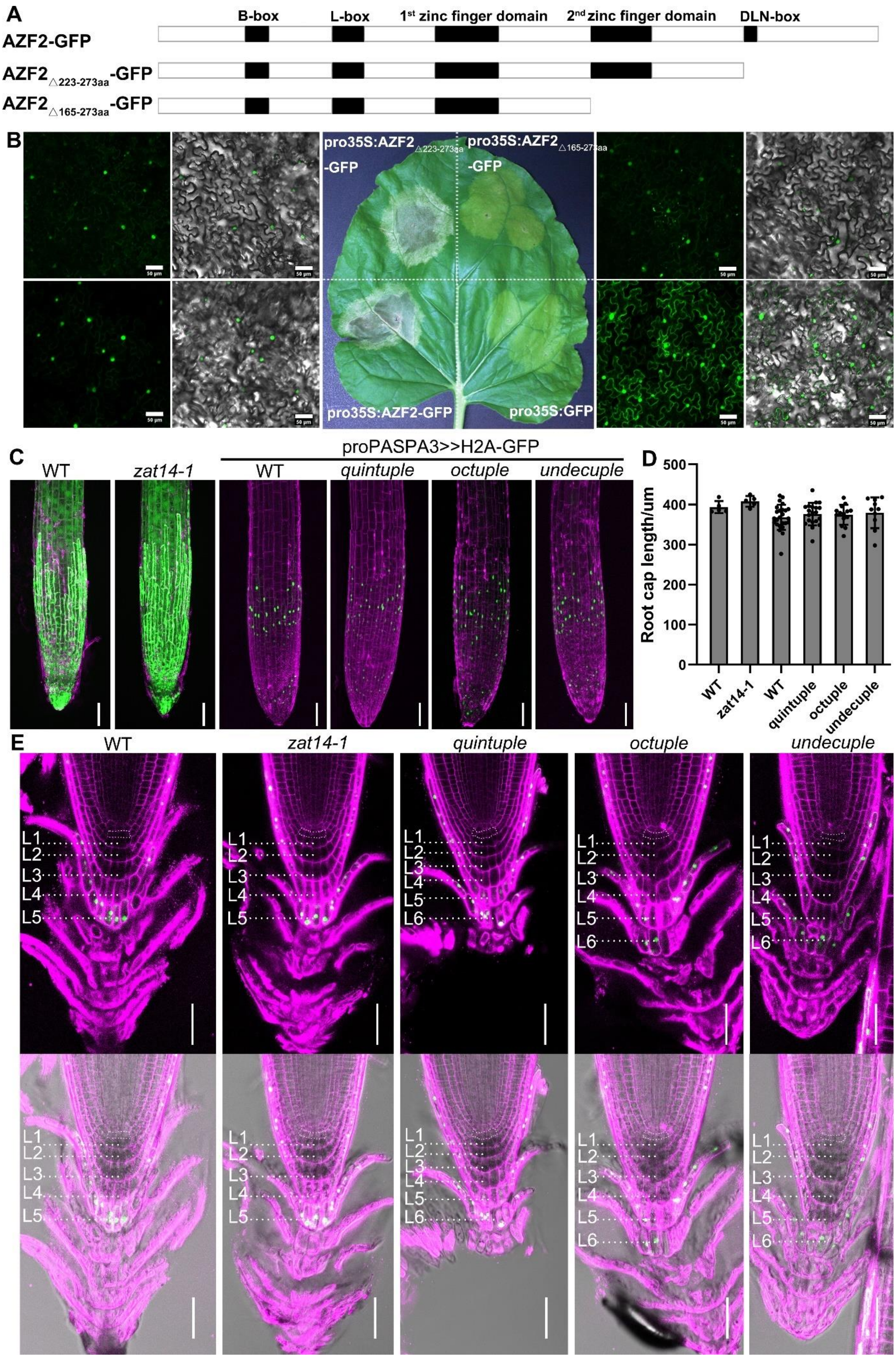

A
B-box
L-box
1st zinc finger domain
2nd zinc finger domain
DLN-box
AZF2-GFP
AZF2△223-273aa-GFP
AZF2△165-273aa-GFP
B
pro35S:AZF2△223-273aa-GFP
pro35S:AZF2△165-273aa-GFP
pro35S:AZF2-GFP
pro35S:GFP
50 μm
C
proPASPA3>>H2A-GFP
WT
zat14-1
WT
quintuple
octuple
undecuple
D
Root cap length/um
500
400
300
200
100
0
WT
zat14-1
WT
quintuple
octuple
undecuple
E
WT
zat14-1
quintuple
octuple
undecuple
L1
L2
L3
L4
L5
L6

Supplemental figure 5. Phenotype analysis of modified versions of AZF2, *zat14-1* single mutant and higher order mutants.

(A) Graphical representation of AZF2 modified versions genomic sequence. AZF2 has several conserved regions, B-box, L-box, two zinc-finger domain and an EAR-motif. In the AZF2$_{\Delta 223\text{-}273aa}$, the EAR-motif domain in the C-terminal has been deleted. In the AZF2$_{\Delta 165\text{-}273aa}$, the gene sequence from the second zinc-finger domain to the C-terminal has been deleted.

(B) Transient overexpression of AZF2 wild type and AZF2$_{\Delta 223\text{-}273aa}$ resulted in ectopic cell death at 7DAI, while the AZF2$_{\Delta 165\text{-}273aa}$ did not result in obvious cell death in *N.benthamiana*. GFP nuclear signal was found in the nucleus at 2 DAI for all examined constructs. pro35S:GFP was used as a control. Bars = 50 µm.

(C) 5-day-old single *zat14* or higher order mutants did not show an abnormal cell death phenotype compared to the wild type. The wild type and the *zat14-1* mutant were stained with FDA (in green) and PI (in magenta) to mark living and dead cells, respectively. The higher order mutants -with *proPASPA3:H2A-GFP* line as background- and the wild type *proPASPA3:H2A-GFP* line were only stained with PI. Each panel consists of two stitched images. Bars = 50 µm.

(D) The root cap length was measured as the distance between the quiescent center and the last PI positive cells from the LRC, and it did not significantly differ between neither the wild-type and the single *zat14* mutant nor the higher order mutants and the *proPASPA3:H2A-GFP* reporter line. Results shown are means ± SD (n = 5 for WT, n = 5 for *zat14-1*, n = 25 for *proPASPA3:H2A-GFP* reporter line, n = 20 for quintuple mutant, n = 14 for octuple mutant, n = 10 for undecuple mutant).Statistical analysis: *t* test; there is not significant difference between each other.

(E) From left to right: representative median sections of root tips from proPASPA3:H2A>>GFP in the wild type, *zat14-1*, ZAT higher order mutants (stained with PI, in magenta). Living columella layers are marked. White dotted box indicate columella stem cells.

Related to Figure 6.
