# Supplementary material for: Repressive C2H2 zinc finger ZAT proteins promote programmed cell death in the Arabidopsis columella root cap
Source: Plant Physiol. Author manuscript; Available in PMC 2023 Nov 30. (PMC10231456; doi:10.1093/plphys/kiad130)
Supplement: Movie Legends [file EMS171097-supplement-Movie_Legends.docx]

**Supplemental movie 1. Confocal time course of ZAT14-GFP inducible overexpression line**

Induced root of *proHTR5:XVE>>ZAT14-GFP* showed ectopic cell death indicated by occurrence of intense PI staining in the transition and elongation zone, finally leading to a stop of root growth. GFP signal is shown in green and PI signal is shown in magenta. Imaging started 6 h after estradiol induction, and roots were imaged every 30 min and followed for approximately 18 h. Bar = 100 µm.

Related to Figure 1.

**Supplemental movie 2. Confocal time course of NLS-GFP inducible line**

The induced control line, *proHTR5:XVE>>NLS-GFP*, showed normal root growth. GFP signal is shown in green and PI signal is shown in magenta. Imaging started 6 h after estradiol induction, and roots were imaged every 30 min and followed for approximately 18 h. Bar = 100 µm.

Related to Figure 1.
